# What Binarization Method Is the Best for Amplitude Inline Fresnel Holograms Synthesized for Divergent Beams Using the Direct Search with Random Trajectory Technique?

**DOI:** 10.3390/jimaging9020028

**Published:** 2023-01-27

**Authors:** Andrey S. Ovchinnikov, Vitaly V. Krasnov, Pavel A. Cheremkhin, Vladislav G. Rodin, Ekaterina A. Savchenkova, Rostislav S. Starikov, Nikolay N. Evtikhiev

**Affiliations:** Laser Physics Department, Institute for Laser and Plasma Technologies, National Research Nuclear University MEPhI (Moscow Engineering Physics Institute), Kashirskoe Shosse 31, 115409 Moscow, Russia

**Keywords:** holography, binarization, optical reconstruction, digital micromirror device, diffractive optical element, computer-generated hologram, thresholding, direct search, error diffusion, Gerchberg–Saxton algorithm

## Abstract

Fast reconstruction of holographic and diffractive optical elements (DOE) can be implemented by binary digital micromirror devices (DMD). Since micromirrors of the DMD have two positions, the synthesized DOEs must be binary. This work studies the possibility of improving the method of synthesis of amplitude binary inline Fresnel holograms in divergent beams. The method consists of the modified Gerchberg–Saxton algorithm, Otsu binarization and direct search with random trajectory technique. To achieve a better quality of reconstruction, various binarization methods were compared. We performed numerical and optical experiments using the DMD. Holograms of halftone image with size up to 1024 × 1024 pixels were synthesized. It was determined that local and several global threshold methods provide the best quality. Compared to the Otsu binarization used in the original method of the synthesis, the reconstruction quality (MSE and SSIM values) is improved by 46% and the diffraction efficiency is increased by 27%.

## 1. Introduction

A digital micromirror device is a microelectromechanical system that forms an image through a micromirror array [1]. Each micromirror corresponds to a pixel of the displayed image. DMD is very perspective device due to its high frame rate. Nowadays, DMD can display up to about 30 thousand images per second [2]. DMDs can be used in various tasks: holographic projection and 3D television [3,4,5], media characterization [6], compressing imaging [7], 3D printing [8], mode generation [9], spectroscopy [10], digital [11] and computer-generated [12] hologram reconstruction, information packaging [13], etc. The DMD operating principle allows for imaging of only binary images. Therefore, binarization is mandatory prior to displaying an image on DMD. Binarization is a technique of converting a halftone distribution into a binary one. This leads to decrease in the performance of diffractive optical elements (DOEs). An important task is to find a binarization method which leads to a lesser decrease of characteristics such as reconstructed image quality and diffraction efficiency.

DOEs are often used for optical formation of object images. This can be achieved by displaying DOE onto DMD while illuminating it with reference beam. A popular type of DOE is the hologram, which can be obtained both through optical [14] and numerical [15] methods. Usually, a hologram is the interference pattern which is formed by the reference and object beams. As a result, carrier spatial frequency is formed. This leads to the appearance of unwanted zero diffraction order. In most cases, a reconstructed object image should be spatially separated from undesirable diffraction orders [16]. The holograms are characterized by low diffraction efficiency (DE) due to the presence of these orders [17].

Phase holograms [18,19,20] allow for higher DE value than their amplitude counterparts. This type of hologram is commonly synthesized by iterative methods such as the Gerchberg–Saxton algorithm [21]. Phase holograms are reconstructed using phase liquid crystal spatial light modulators [22]. Since these holograms are phase modulated, they cannot be directly displayed onto fast amplitude DMDs.

To change this, an iterative method of synthesis of amplitude binary inline Fresnel holograms in divergent beams was recently proposed [23]. It consists of three components: the modified Gerchberg–Saxton algorithm, Otsu binarization [24] and direct search with random trajectory technique (DSRT) [25,26] at the end of the method. The application of DSRT allows to substantially increase reconstruction quality and DE. However, this final step of the method is not mandatory, while the binarization step is necessary. However, only the Otsu global thresholding [24] was applied in the past. There are groups of binarization methods that can potentially provide better results [27,28,29,30,31].

The aim of this work is improving the method of synthesis of binary inline Fresnel holograms by applying the optimal binarization technique. This is achieved by conducting a comparative analysis of the quality of image reconstruction and diffraction efficiency of holograms synthesized using the new technique and binarized with different methods. Analysis included numerical and optical experiments on the reconstruction of binarized holograms both before and after application of DSRT technique.

The rest of this paper is organized as follows. In Section 2, the investigated techniques are described: the method of synthesis of binary inline Fresnel holograms and binarization methods. In Section 3, parameters to compare, numerical experiments and their results are given. The optical experiments and results are presented in Section 4. The main results are provided in the Conclusions.

## 2. The Methods

### 2.1. The Method of Synthesis of Binary Inline Fresnel Holograms

The method of synthesis of binary inline Fresnel holograms allows for image reconstruction in divergent beams using DMD [23]. The method consists of three stages. The first stage is a modified iterative Gerchberg−Saxton algorithm. The first modification is that the algorithm operates with the amplitude, and not the phase, in the hologram plane, thus enabling iterative synthesis of amplitude holograms. In classic Gerchberg−Saxton algorithm [21], the hologram is phase one [18,19,20]. The second modification consists of the application of divergent illuminating beam which effectively negates harmful impact of zero and minus first diffraction orders by dispersing them across large area, thus enabling inline hologram geometry with single focused diffraction order. In the Gerchberg−Saxton algorithm, the illumination beam is a plane one. The first stage of the method can be described as follows:

- As a first approximation of the DOE, random amplitude is generated. It is multiplied by the amplitude and phase of a spherical wavefront of a given curvature.
- The Fresnel transform is applied to get into object plane.
- The amplitude of the obtained distribution is replaced by the required one, and the phase remains unchanged.
- An inverse Fresnel transform is applied to get into the hologram plane.
- The phase of the obtained distribution is replaced by a spherical one, and the amplitude remains unchanged.

These steps are repeated until the required number of iterations are completed, which is determined via target function value stagnation. The target function is the superposition of two parameters: quality metrics (for example, normalized standard deviation, NSTD) and DE.

Then, the second stage takes place. The obtained halftone DOE is binarized. In [23], only the Otsu threshold method was used. The question of using other binarization methods was not considered.

The third stage is aimed at reducing the synthesis error based on the results of the first stage. For this, the DSRT technique is used [25,26]. Every hologram pixel is consequently changed in order set by the random trajectory. For the binary DOEs, an element with a zero value is switched to one, and an element with non-zero value is switched to zero. If the synthesis error is reduced, the change remains; otherwise, it is canceled. At the end of the process, we display the resulting binary hologram on the DMD and register the optically reconstructed object image with a camera.

The three stages have different calculation times. The third stage lasts extremely long—depending on the hologram size and PC performance, it can last from minutes to days. The first two stages last several dozen seconds at most. Since, in practical application, there is a need to reduce the hologram synthesis time, the choice of the optimal binarization method can drastically reduce the calculation time of the third stage.

### 2.2. Binarization Methods

Nowadays, the image binarization procedure is actively used for various tasks: for example, for character recognition on damaged [32] and scanned historical documents [33], for correction of inhomogeneously illuminated texts [34], etc. Moreover, binarization is an important technique for high-speed binary DMD applications [1].

In the method of synthesis of amplitude binary inline Fresnel holograms, binarization is performed after the first stage. This allows one to begin the final DSRT stage. Choosing the optimal binarization method allows the improvement of the method: to reduce synthesis error, increase diffraction efficiency and/or reduce calculation time. Popular and widely used binarization methods are divided into groups: global [24,35,36,37,38,39,40,41,42,43,44,45] and local [46,47,48,49,50,51] thresholding, standard [52,53,54] and dot [29,55,56,57] error diffusion, and iterative techniques [58,59]. In the global thresholding, parameters (global mean and variance) are calculated using all pixel values. The image is obtained through binarization using a single threshold. The following global thresholding methods that demonstrated the best quality of the binarized DOE are those of: Otsu [24], Li [35], Kittler [36], Ridler [37], Huang [38], Prewitt (Pr1, Pr2) [39], Kapur (Kapur 1, Kapur 2) [40], Glasbey (Glas) [41], Tsai [42], Doyle [43], Shanbhag (Shan) [44], and Yen [45]. For local thresholding, the parameters (local mean, mean deviation, etc.) are calculated for individual windows. The image is obtained via binarization using a number of thresholds. Almost all local thresholding methods showed good results for DOE binarization. They are: Niblack [46], Sauvola [47], Zhang (ZhTa) [48], Nick [49], Wolf (Wol) [50], Mean [34], Median [34], MidGrey (MidGr) [34] and Phansalkar (Phan) [51]. Iterative methods use the dividing of intensity histogram into separate segments. Thresholds are calculated for each segment in the Shaikh method (Sh) [58].

For the error diffusion, the pixel value is compared with some threshold value (for example, half the maximum, the median value, the average, etc.). If the pixel value is lower than the threshold, it is assigned to a zero; otherwise, to one. Difference between obtained value and threshold is an error. This error is distributed to neighboring pixels in accordance with the weighting matrix (weighting coefficients; kernel of error diffusion). Direction and order of bypass of pixels have an effect on binarization quality. In the case of standard weighting matrix, already-processed pixels do not participate in the further error propagation. Only a small number of error diffusion techniques showed good results in preliminary experiments. Thus, only the most popular methods were added to the final comparison: Floyd–Stenberg (Fld) [52], Jarvis (Jrv) [53], and Atkinson (Atk) [54]. For dot diffusion, the error value is propagated among all neighbors of the considered pixel. Thus, already-processed pixels participate in the further propagation of the error value. The methods of Knuth (Kn) [55], Guo (Guo) [56], Dot variative coefficients (Dvc) [29], Liu with coefficients 24 (L24), and 50 (L50) [57], and Cheremkhin (Ch) [29] were added to the comparison.

## 3. Numerical Experiments

### 3.1. Numerical Experiment Conditions

**Figure 1:**
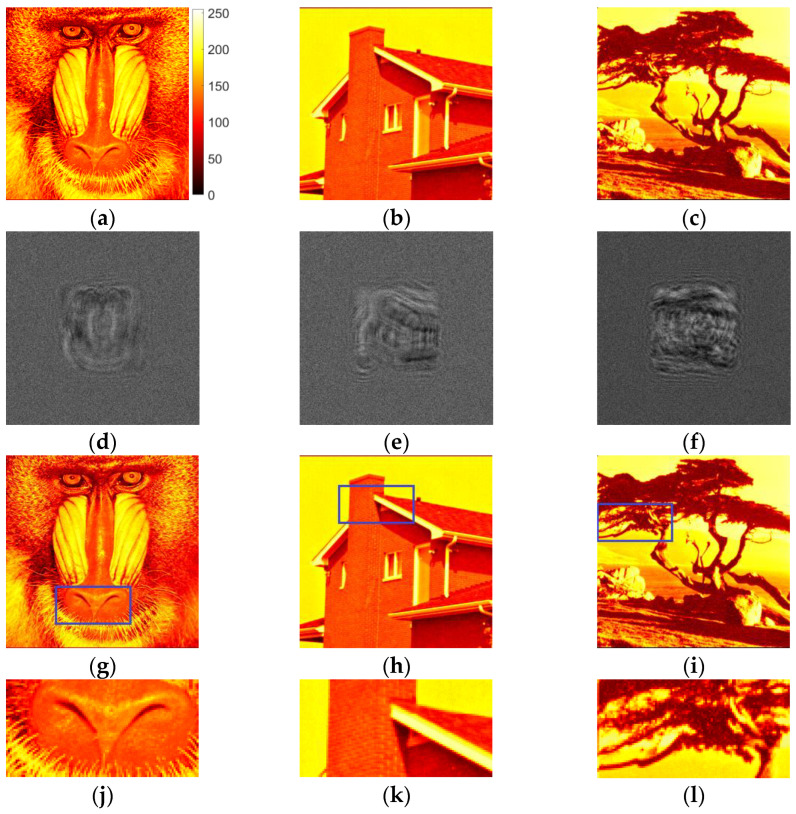
Test images (**a**–**c**), synthesized halftone amplitude inline Fresnel holograms (**d**–**f**), reconstructed images (**g**–**i**) and their enlarged fragments (**j**–**l**).

We performed numerical experiments on the synthesis of amplitude binary Fresnel holograms for the reconstruction in divergent beams. Halftone images of up to 256 × 256 pixels were used as the test objects. They are shown in Figure 1a–c.

Initially, the first stage of the method of synthesis of holograms was performed. A modified Gerchberg–Saxton method was used. Examples of obtained halftone (8 bit) holograms with size of 1024 × 1024 pixels are shown in Figure 1d–f. The reconstructed images are given in Figure 1g–i. For more accurate image comparison, the reconstructed images (Figure 1g–i) were normalized so that the mean value of each image is equal to the mean value of original image (Figure 1a–c). In Figure 1j–l, enlarged fragments of the reconstructed images are shown. The positions of these fragments on the original images are highlighted with rectangles in Figure 1g–i. For better visualization, object images are shown in color graphic representation instead of grayscale one. Color bar for these and below (Section 3.3 and Section 4) images are provided in Figure 1a. The “focal length” was equal to 0.3 m. It is the radius of curvature of the wavefront set during the hologram synthesis (see Section 2.1). The inline type of the synthesized holograms provides scattering of zero diffraction order across large area in the image reconstruction plane.

Next, binarization methods were applied to the obtained halftone hologram. The quality of reconstruction and DE were evaluated.

In order to analyze the possibility of further improvement of binary DOEs, the DSRT technique was used. We used five bypasses of all pixels of the hologram. The reconstructed image is analyzed after each pixel signal changing. The quality of reconstructed images and DE were also evaluated.

### 3.2. Error Metrics

Three metrics were used to analyze the binary holograms and corresponding reconstructed images. The structural similarity index (SSIM) aims to evaluate the quality of the image *B*(*x*,*y*) relative to the original one *A*(*x*,*y*). The SSIM analyzes brightness *l(A*,*B*), contrast *c*(*A*,*B*) and structure *s*(*A*,*B*) of the images [60]:

(1) $$SSIM=l^{\alpha }\cdot c^{\beta }\cdot s^{\gamma }={\left(\frac{2\mu_{A}\mu_{B}+C_{1}}{\mu_{A}^{2}+\mu_{B}^{2}+C_{1}}\right)}^{\alpha }\cdot {\left(\frac{2\sigma_{A}\sigma_{B}+C_{2}}{\sigma_{A}^{2}+\sigma_{B}^{2}+C_{2}}\right)}^{\beta }\cdot \left(\frac{\sigma_{AB}+C_{3}}{\sigma_{A}\sigma_{B}+C_{3}}\right),$$

where *μ_A_*, *μ_B_*, *σ_A_*, *σ_B_*, and *σ_AB_* are local means, standard deviations and cross covariance for the images *A*(*x*,*y*) и *B*(*x*,*y*); *C*_1_, *C*_2_ and *C*_3_ are regularization constants for brightness, contrast and structural parameters; *α*, *β* and *γ* are usually equal to one. SSIM is equal to values between −1 and 1. The closer the reconstructed image to the original, the closer SSIM to 1.

Diffraction efficiency (DE) is the part of radiation power that forms object image relative to the incident on the hologram power. DE is the product of average hologram transmission *T* and the fraction of the first diffraction order relative to sum of all orders *O*:

(2) $$DE=T\cdot O=T\cdot \frac{I_{obj}}{I_{all}}$$

where *I_obj_*—intensity of the object image; *I_all_*—intensity of all diffraction orders. The higher the DE value, the brighter the reconstructed object image.

The mean squared error (MSE) estimates the effect of image degradation due to processing, noises, quantization, etc. It is calculated for normalized original and reconstructed images as follows [32]:

(3) $$MSE=\frac{\sum_{x,y=1}^{M,N}{\left(A\left(x,y\right)-B\left(x,y\right)\right)}^{2}}{M\cdot N}$$

where *M*, *N*—the quantity of rows and columns of pixels of the images. The lower the MSE value, the lesser the effect of degradation factors on the image.

Obtained SSIM, DE and MSE values for halftone holograms (see Figure 1) are shown in Table 1.

It can be seen that the quality of reconstruction is very good. This is due to a large quantity of halftone gradations. Decrease of gradations to several or even two results in drastic degradation of image quality.

### 3.3. Results of Numerical Experiments

**Figure 2:**
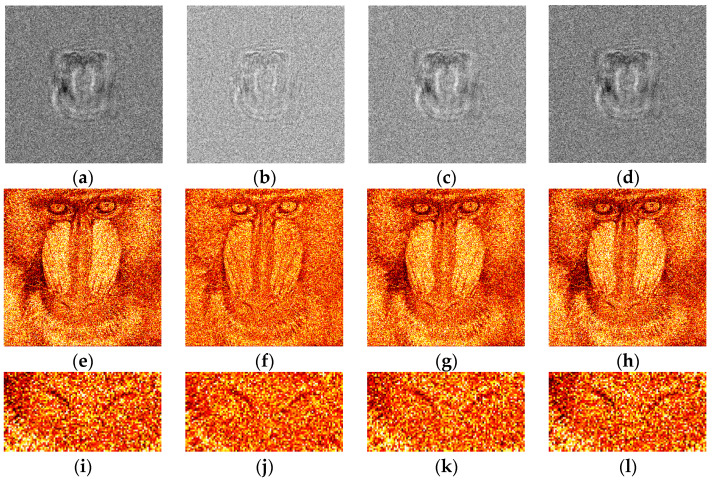
Examples of synthesized binary inline Fresnel holograms containing the “Baboon” image, corresponding reconstructed images and their enlarged fragments. Holograms were binarized using the Otsu (**a**,**e**,**i**), Sauvola (**b**,**f**,**j**), Li (**c**,**g**,**k**), and Huang (**d**,**h**,**l**) methods.

**Figure 3:**
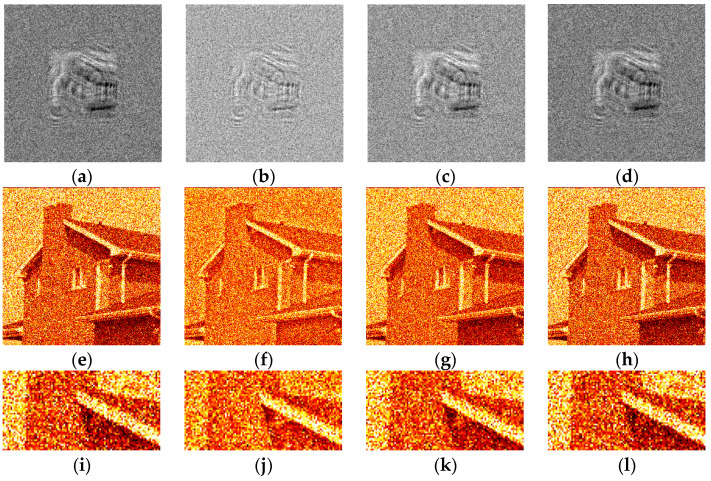
Examples of synthesized binary inline Fresnel holograms containing the “House” image, corresponding reconstructed images and their enlarged fragments. Holograms were binarized using the Otsu (**a**,**e**,**i**), Sauvola (**b**,**f**,**j**), Li (**c**,**g**,**k**), and Huang (**d**,**h**,**l**) methods.

**Figure 4:**
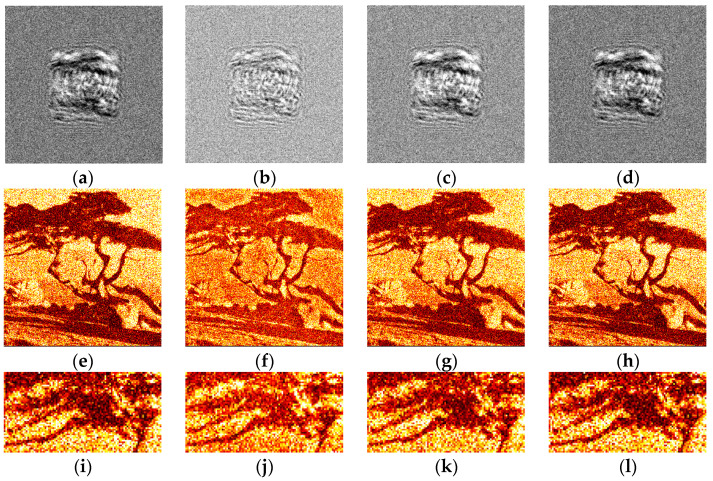
Examples of synthesized binary inline Fresnel holograms containing the “Tree” image, corresponding reconstructed images and their enlarged fragments. Holograms were binarized using the Otsu (**a**,**e**,**i**), Sauvola (**b**,**f**,**j**), Li (**c**,**g**,**k**), and Huang (**d**,**h**,**l**) methods.

The synthesized amplitude inline Fresnel holograms were binarized with various methods. Examples of the holograms after binarization using the Otsu method are given in Figure 2a, Figure 3a and Figure 4a. Corresponding reconstructed images and their enlarged fragments are shown in Figure 2e,i, Figure 3e,i and Figure 4e,i. For comparison, holograms after binarization using several best methods are shown. These methods are those of Sauvola (Figure 2b,f,j, Figure 3b,f,j and Figure 4b,f,j), Li (Figure 2c,g,k, Figure 3c,g,k and Figure 4c,g,k), and Huang (Figure 2d,h,l, Figure 3d,h,l and Figure 4d,h,l). The Sauvola method provides better line elements than the Otsu, Li, and Huang methods. It can be seen that in Figure 2j, (Sauvola method) the border lines (nostrils) are more clearly presented. In addition, the lines (white hair) are thinner, whereas in Figure 2i,k,l they are not visible at all. In Figure 3j (Sauvola method), the window located under the roof is visible unlike in the case of Otsu and Huang methods. In Figure 3k (Li method), it is visible but significantly worse. In Figure 4j (Sauvola method), boundaries between dark branches and light background are sharper than in case of the Otsu, Li, and Huang methods. The SSIM value for the Sauvola method is equal to 0.23, which is 21% better than the Otsu method provides. The MSE is equal to 0.12, which is 30% better than the Otsu method obtained. The quality of reconstruction can be further significantly improved by the third DSRT stage (end of the Section 3.3).

**Figure 5:**
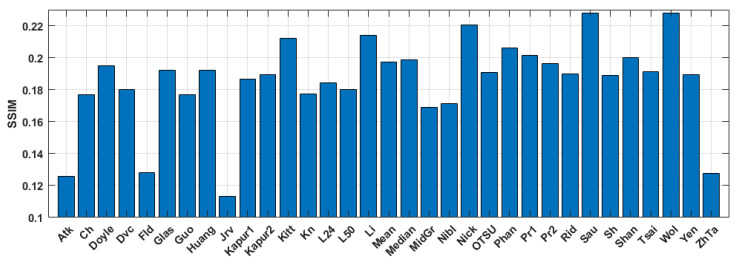
Average SSIM values for the binary amplitude inline Fresnel holograms.

**Figure 6:**
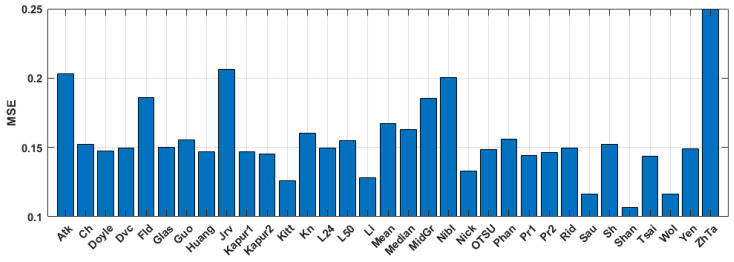
Average MSE values for the binary amplitude inline Fresnel holograms.

**Figure 7:**
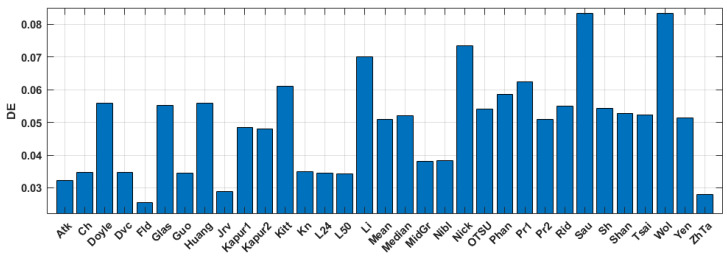
Average DE values for the binary amplitude inline Fresnel holograms.

The quantitative evaluation of the results is shown in Figure 5, Figure 6 and Figure 7. SSIM, DE, and MSE values for the best 33 binarization methods are presented. It can be seen that many local and several global thresholding methods provide the best average results. The best methods are: local Sauvola, Wolf, Nick, and global Li. Additionally, good results were demonstrated by the Phansalkar, Kittler, Prewitt, Doyle, and Huang methods.

Changing the trajectory for error diffusion methods has a little effect on the reconstruction. This is due to the fact that original object images are halftone. Dot diffusion provides satisfactory SSIM and MSE values. However, DE is significantly lower compared to what the thresholding methods obtained. In result, the error diffusion is not well-suited for the third stage of the method of synthesis of amplitude inline Fresnel holograms. Target function is a superposition of NSTD and DE. Since DE is already low, for these methods, degrees of freedom to reduce the target function are limited. Thresholding methods with higher DE can provide better quality in the third stage.

**Figure 8:**
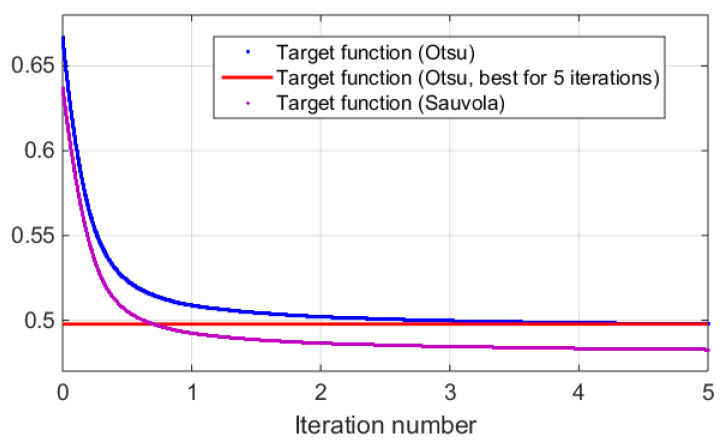
Dependency of target function vs. iteration number during DSRT stage of hologram synthesis after binarization using the Otsu and Sauvola methods.

Dependencies of target function during the third stage of synthesis of the holograms vs. iteration number are shown in Figure 8. A single iteration is a go-round of all pixels of the hologram. Consequently, it consists of many pixel value changes and can be fractional. For comparison, two methods of hologram binarization were used: Otsu, and Sauvola ones. The contributions of NSTD and DE were equal in the target function. The object image is “Baboon” (see Figure 1a). For better demonstration of the results, line corresponding to the best Otsu method value (i.e., for all 5 iterations) is also given. It can be seen that 0.7 iterations (see data point of intersection of a Sauvola method target function with a best Otsu method line) of DSRT were enough for the “Baboon” object to exceed the quality that was obtained for the Otsu binarization method with 5 DSRT iterations. For other objects, the dependencies are similar.

**Figure 9:**
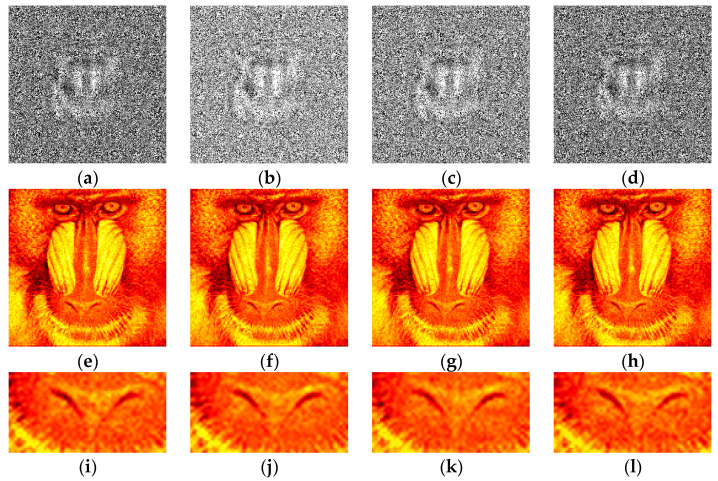
Examples of synthesized binary inline Fresnel holograms containing the “Baboon” image after application of DSRT technique, corresponding reconstructed images and their enlarged fragments. Holograms were binarized using the Otsu (**a**,**e**,**i**), Sauvola (**b**,**f**,**j**), Li (**c**,**g**,**k**), and Huang (**d**,**h**,**l**) methods.

**Figure 10:**
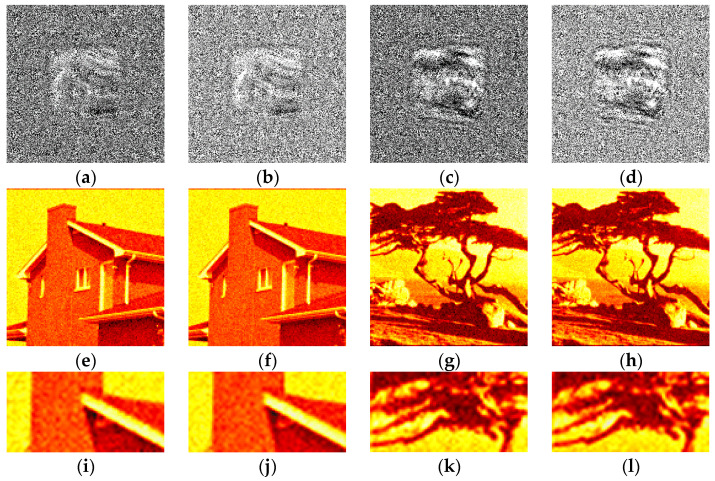
Examples of synthesized binary inline Fresnel holograms containing the “House” and the “Tree” images after application of DSRT technique, corresponding reconstructed images and their enlarged fragments. Holograms were binarized using the Otsu (**a**,**e**,**i** and **c**,**g**,**k**), and Sauvola (**b**,**f**,**j** and **d**,**h**,**l**) methods.

Examples of synthesized binary inline Fresnel holograms after DSRT stage and corresponding reconstructed images are shown in Figure 9 and Figure 10. The Otsu and the best method (Sauvola) are shown in Figure 9a,e,i, Figure 10a,e,i and Figure 10c,g,k and Figure 9b,f,j, Figure 10b,f,j and Figure 10d,h,l correspondingly. For comparison, Li (Figure 9c,g,k), and Huang (Figure 9d,h,l) methods are also shown. The images in Figure 9 and Figure 10 are similar from the visual point of view. However, the trend of sharper boundaries between object and background remains for the Sauvola method compared to the Otsu method. This plays a positive role in combination with a higher DE for the Sauvola method. Quality and diffraction efficiency of synthesized binary holograms are shown in Table 2.

It can be seen that the Otsu method provides good quality of reconstruction. However, for all halftone images, the Sauvola method provided better quality of up to more than 60%. A fraction of single iteration or several iterations of the DSRT after the Sauvola method may be enough to exceed the reconstruction quality compared to the Otsu method with all 5 DSRT iterations. 

It can be seen that several other methods provide better quality than the Otsu also. For example, another global thresholding method—Li—provides a significantly lower MSE value (see Table 2).

To additionally verify the obtained results, the binarized holograms were displayed onto DMD and optically reconstructed.

## 4. Optical Experiments

**Figure 11:**
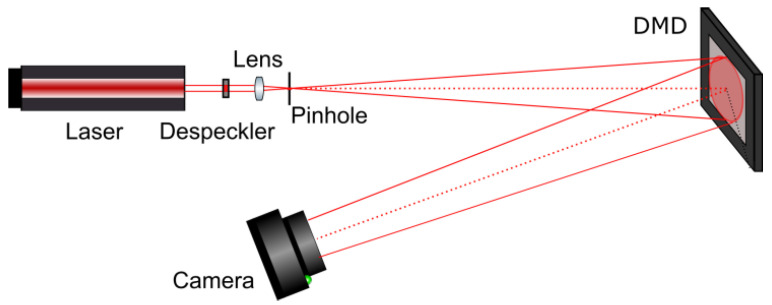
Scheme of the experimental setup for hologram image reconstruction using DMD in divergent beams.

The synthesized amplitude binary inline Fresnel holograms were optically reconstructed using the experimental setup shown in Figure 11. He–Ne laser (633 nm, 10 mW) was used as a light source. Despeckler Optotune LSR-3010 decreased spatial coherence of the beam. Lens and pinhole forms spherically diverging beam required for holograms’ reconstruction. Divergent light beam falls onto DMD DLP9500BFLN (1920 × 1080 pixels, pixel size 10.8 × 10.8 μm, framerate up to 23 kHz) used for holograms displaying. Retiga R6 camera (CCD sensor, 2688 × 2200 pixels, pixel size 4.6 × 4.6 µm) registered optically reconstructed images. 

**Figure 12:**
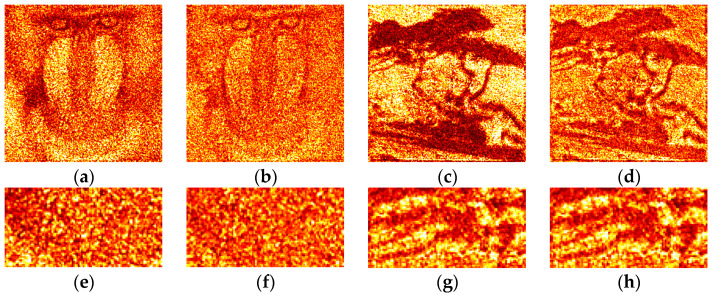
Examples of optically reconstructed images. The holograms were binarized using the Otsu (**a,e** and **c**,**g**) and Sauvola (**b**,**f** and **d**,**h**) methods.

**Figure 13:**
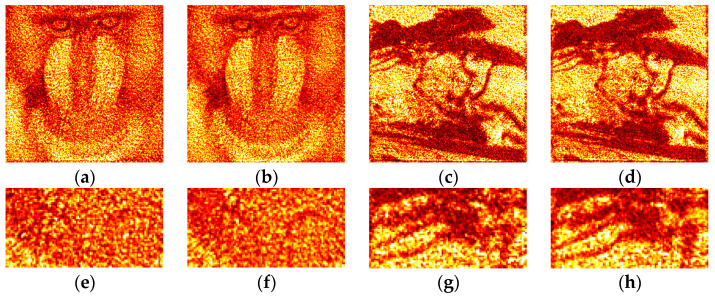
Examples of optically reconstructed images. The holograms were binarized using the Otsu (**a,e** and **c**,**g**) and Sauvola (**b**,**f** and **d**,**h**) methods with additional DSRT technique.

Examples of optically reconstructed images are shown in Figure 12 and Figure 13. The Otsu (a,e and c,g) and Sauvola (b,f and d,h) binarization methods were used. In Figure 12, holograms were reconstructed just after binarization (i.e., after the second stage of the synthesis method). In Figure 13, holograms were reconstructed after application of additional DSRT technique (i.e., after third stage). We can see that the DSRT stage significantly improved the image quality. The object image became less noisy. However, the difference between images in the Figure 13 can also be seen. For example, borders (Figure 13f) and lines (Figure 13h) are clearer and sharper than in the Otsu case (Figure 13e,g).

Reconstructed images were quantified. Average improvement of the images after hologram binarization using the Sauvola method is 62% (SSIM), 27% (DE), and 29% (MSE) compared with the Otsu method. After employing the DSRT technique, the Sauvola method provides better SSIM (on 61%), MSE (on 11%), and DE (on 5%). Results are shown in Table 3. The DE of the optically reconstructed holograms was compared with the Otsu or Otsu-based DSRT method. These relative DEs are shown in the Table 3. Quality metrics for optical experiments are lower than in the case of numerical ones. This is mainly due to the presence of speckle noise, defects of the optical path and the DMD.

Thus, choosing the optimal binarization method can significantly improve the image quality and DE. This optimal binarization provides substantial results’ improvement already. DSRT stage provides further quality improvement. To achieve the same hologram quality using optimal binarization technique, about a single DSRT iteration is required instead of the five needed with Otsu binarization (for example, see Figure 8). Thus, we achieved a five-fold increase in speed of calculations without any loss of quality.

## 5. Conclusions

In this paper, we analyzed the possibilities of improving the method of synthesis of binary amplitude inline Fresnel holograms. Global and local thresholding, dot and standard error diffusion binarization methods were compared.

Object images were reconstructed from holograms in numerical and optical experiments. Many local (e.g., Sauvola, Wolf, Nick) and several global (e.g., Li and Kittler) threshold methods can be considered the best binarization methods. The results of optical reconstruction are in agreement with the results of numerical simulations. Compared to the Otsu binarization used in the second stage of the original synthesis method, the reconstruction quality (MSE and SSIM values) is improved by 46%. The diffraction efficiency is increased by 27%. The use of the optimal binarization method significantly improves the reconstructed image quality and diffraction efficiency. 

With use of optimal binarization method, the DSRT stage improves hologram quality by 36% compared to the Otsu-based DSRT. Alternatively, an increase of up to five times can be achieved in calculation speed without loss of quality compared to the Otsu-based DSRT.

The results can be used in processing, high-speed visualization and beam shaping using DMD and binary holograms.

## Figures and Tables

**Table 1:** Quality and diffraction efficiency of the halftone holograms.

Object	Baboon	House	Tree
SSIM	1.0	0.999	0.999
DE	0.047	0.050	0.045
MSE, 10^−4^	5.8	4.8	14.7

**Table 2:** Quality and diffraction efficiency of the numerically reconstructed binary holograms.

Object	Baboon				House		Tree	
Method	Otsu	Sauvola	Li	Huang	Otsu	Sauvola	Otsu	Sauvola
SSIM	0.87	0.90	0.89	0.87	0.71	0.75	0.85	0.87
DE	0.063	0.086	0.077	0.065	0.063	0.084	0.064	0.082
MSE, 10^−3^	3.6	3.4	2.0	3.3	1.7	1.3	2.1	1.3

**Table 3:** Quality and diffraction efficiency of the optically reconstructed binary holograms.

Stage	Binarization				DSRT			
Method	Baboon		Tree		Baboon		Tree	
	Otsu	Sauvola	Otsu	Sauvola	Otsu	Sauvola	Otsu	Sauvola
SSIM	0.093	0.151	0.107	0.174	0.099	0.171	0.124	0.185
DE_rel_	1	1.33	1	1.22	1	1.03	1	1.07
MSE, 10^−2^	16.2	14.7	14.8	10.1	12.7	11.5	11.8	10.7

## Data Availability

Not applicable.
